# Modification of Uniaxial Stress–Strain Model of Concrete Confined by Pitting Corroded Stirrups

**DOI:** 10.3390/ma17236014

**Published:** 2024-12-09

**Authors:** Zhiwei Miao, Yifan Liu, Kangnuo Chen, Xinping Niu

**Affiliations:** School of Civil Engineering, Southeast University, Nanjing 211189, China; kangnuo1212@163.com (K.C.); 220231385@seu.edu.cn (X.N.)

**Keywords:** pitting corrosion, probabilistic model, tension stiffening, confined concrete, revised model

## Abstract

To investigate the impact of stirrup pitting corrosion on the stress–strain model of core concrete under compression, this study, based on existing corroded steel specimens, establishes a probabilistic model of the residual cross-sectional area distribution of steel bars to reasonably evaluate the effect of pitting on the mechanical performance of stirrups. Considering the tension stiffening effect in reinforced concrete, a time-dependent damage model of corroded steel bars in concrete was determined, and the existing stress–strain model of concrete confined by stirrups was ultimately modified, establishing a time-dependent constitutive model that incorporates the effects of stirrup pitting corrosion. A comparison with previous experimental results indicates that the revised model presented in this paper can appropriately reflect the changes in the mechanical performance of concrete confined by corroded stirrups. The results of this study can provide theoretical support for the refined numerical analysis of reinforced concrete structures under the erosion of chloride ions.

## 1. Introduction

With the widespread use of reinforced concrete (RC) structures globally, civil engineers are now confronting an increasing volume of aged civil structures and infrastructure, among which corroded RC buildings have been attracting considerable attention [1,2,3,4]. The corrosion of steel reinforcements can result in detrimental effects including spalling of the concrete cover [5,6,7], bond-slip between the steel and concrete [8], reduced load-bearing capacity of the longitudinal reinforcement [9,10,11,12], and the diminished confinement efficiency of the stirrups on the core concrete [13,14]. These issues significantly impair the seismic performance of RC components/structures and seriously threaten their safety and reliability.

In corrosive environments, stirrups are more prone to erosion than longitudinal reinforcements since they are positioned closer to the concrete cover [15,16]. Previous research and field investigations have reported that under the same corrosive conditions, stirrups tend to exhibit a greater degree of corrosion than longitudinal bars [17,18,19]. Severe corrosion of the stirrups leads to a significant deterioration in their mechanical properties, reducing their effective confinement of the core concrete [20,21]. According to classical constitutive models for core concrete [22,23], the enhancement in deformation capacity provided by stirrup confinement is considerably greater than the improvement in load-bearing capacity. When the stirrups become corroded, the deformation capacity of the core concrete is substantially reduced, which decreases the ductility of the structural components and increases their susceptibility to brittle failure, potentially causing the collapse of structures in seismic zones [24,25,26].

Advanced numerical models have been developed to predict the mechanical performance of corroded RC elements, aiming to capture the effects of corrosion on RC components under compression [24,27,28], bending [2,20,29], or shear loads [26,30,31]. However, the accuracy of these numerical models relies heavily on the reliability of the model parameters of the material, including the residual properties of the corroded longitudinal reinforcement and the compressive performance of the core concrete with the corroded stirrups [4,32,33,34,35]. Consequently, establishing an accurate stress–strain confinement model is essential for assessing both the seismic performance and reinforcement strategies for corroded RC elements. To tackle this challenge, there is an urgent need to verify the confinement performance of transverse corroded reinforcements in RC columns.

Numerous researchers have investigated the mechanical behavior of corroded RC columns, particularly focusing on the degradation of seismic and shear performance [36,37,38]. However, studies on the confinement performance of corroded reinforced concrete columns are limited. Zhou et al. [24] tested 39 RC cylinder specimens with stirrups of varied corrosion levels and found that the degradation in mechanical behaviour is more pronounced in low-strength grade-confined concrete specimens compared with those with higher concrete strength grades. Zhang et al. [13] performed compression load tests on different types of corroded RC columns, considering factors including corrosion levels, size effects, stirrup configurations, and the simultaneous corrosion of stirrups and longitudinal reinforcements. They found that the load capacity, stiffness, and ductility of the confined concrete decreased by varying degrees as the level of corrosion increased. Based on the experimental data, semi-empirical and semi-theoretical formulas were proposed for calculating the compressive strength and the corresponding strain. Andisheh et al. [27] utilized estimates of four crucial parameters—maximum compressive stress, strain at maximum compressive stress, concrete’s modulus of elasticity, and ultimate compressive strain—to predict the stress–strain relationship of confined concrete exposed to reinforcement corrosion. Wu et al. [39] discovered that sustained loading could decrease the effective corrosion degree of stirrups, and their results indicated that the ultimate load capacity of columns increased with greater sustained loads. Zhang et al. [21] introduced modification factors in their study to account for the effect of corroded stirrups on the confinement stress and strain of core concrete. Additionally, an existing stress–strain model for confined concrete [23] was revised to more accurately reflect the effects of corroded stirrups. Building on the same model, Vu et al. [40]. further refined the stress–strain model for concrete confined by corroded stirrups through tests on corroded RC columns with various sectional arrangements.

While considerable research has laid a basis for analyzing the degradation mechanism associated with corroded RC columns, most studies have been conducted under the assumption of uniform corrosion. It is generally believed that corrosion induced by carbonation tends to be uniform [41,42], whereas chloride-induced corrosion often causes significant non-uniformity in both the longitudinal and circumferential directions of the reinforcement, leading to pitting corrosion [43,44,45]. Extensive surveys have indicated that completely uniform corrosion is almost non-existent in practical engineering. Compared to uniform corrosion, pitting corrosion significantly diminishes the tensile load-bearing and deformation capacity of the reinforcement [3,9,46]. Therefore, when assessing the performance of RC components and structures, it is crucial to reasonably account for the adverse impact of pitting corrosion on the reinforcement.

Extensive research has been conducted on the residual mechanical properties of individual corroded rebar [9,10,46,47]. It has been reported in the literature [48,49] that the mechanical properties of corroded steel reinforcements can depend on the area ratio of different microstructures after corrosion, such as in the case of quenched and self-tempered (QST) steel bars. However, this effect is relatively small when considering the overall mechanical behaviour. Extensive experimental studies have consistently shown that the ultimate load-bearing capacity of the corroded reinforcement is primarily governed by its minimum residual cross-sectional area [9,10,11,12,46,50]. Specifically, the nominal yield strength and ultimate strength (calculated as the ratio of the tensile force to the original cross-sectional area) have been observed to decrease linearly with increasing corrosion levels. However, the true yield strength and ultimate strength (calculated as the ratio of the tensile force to the actual minimum cross-sectional area) remain unaffected and may even show a slight increase under high corrosion levels [11,12,51,52].

Regarding the deterioration of the deformation capacity of corroded rebars, this is primarily due to the strain localization caused by pitting corrosion. This phenomenon can be effectively addressed using a theoretical model that accounts for various corrosion patterns and a relative length characteristic, enabling it to accurately predict the residual deformation capacity of corroded rebar for different gauge lengths [3]. Despite these advanced developments at the material level, the actual behaviour of corroded rebar in an RC component setting and the coupling effects through the surrounding concrete are always being overlooked.

**Figure 1:**
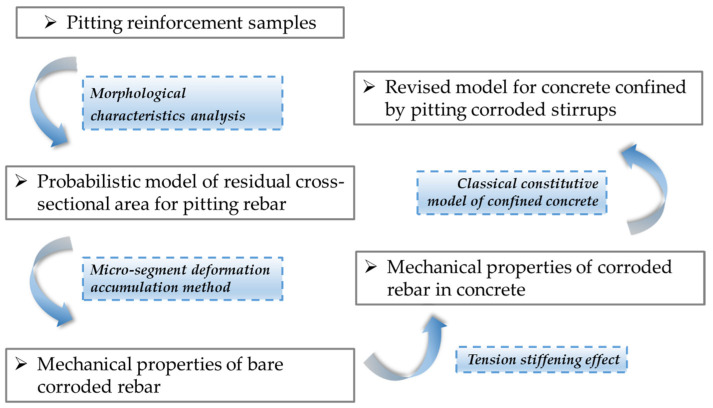
Flowchart of research methodology.

In light of this, this study establishes a probabilistic model to describe the distribution of the residual cross-sectional area of pitted reinforcement samples, aiming to reasonably assess the variability in the residual area resulting from non-uniform corrosion. A micro-segment deformation accumulation method was introduced to evaluate the performance of corroded rebars. By considering the tension stiffening effect in reinforced concrete, a time-dependent damage model for corroded reinforcements within concrete was determined. Subsequently, existing stress–strain models for concrete confined by stirrups were revised to develop a time-dependent constitutive model that incorporates the effects of stirrup pitting corrosion. The reliability of the revised model was verified through comparative analyses with experimental results. The entire research methodology and technical roadmap of this study are depicted in Figure 1.

## 2. Impact of Corrosion on the Tensile Performance of Reinforcement

Over the past few decades, numerous experimental studies have been conducted on the residual mechanical properties of corroded rebar. Most studies have developed regression formulas to describe the connection between corrosion levels and residual mechanical properties based on experimental results. However, variations in research contexts and methodologies adopted by different scholars result in differing observed corrosion morphologies, which in turn lead to variations in the empirical models. These discrepancies among the different degradation models pose significant challenges for subsequent applications. The choice of different degradation models can substantially impact the assessment results, including evaluating core concrete performance and conducting numerical analyses of RC structures. Therefore, properly accounting for the impact of non-uniform corrosion on the mechanical properties of reinforcements is a prerequisite for accurately assessing the compressive performance of core concrete.

### 2.1. Investigation of Non-Uniform Corrosion Morphological Features

**Figure 2:**
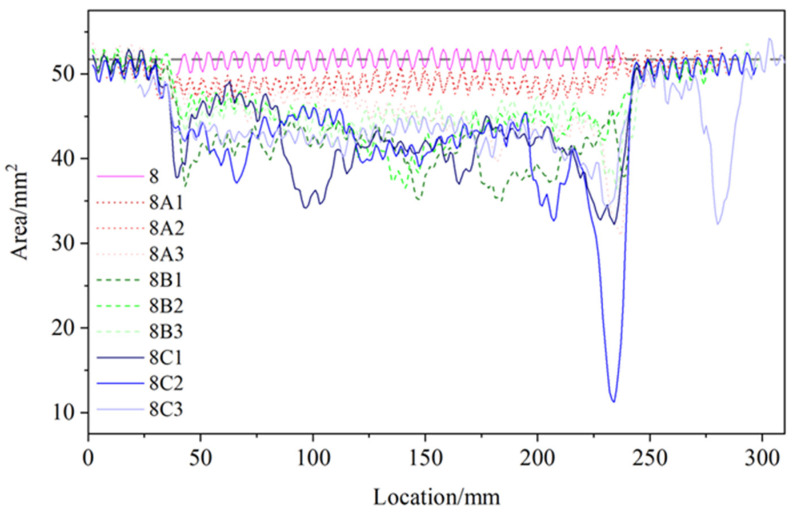
Spatial distribution of remaining cross-sectional areas of 8 mm corroded rebars along the longitudinal direction [9].

To establish a connection between the morphological characteristics of non-uniformly corroded reinforcements and their residual mechanical properties, our group [9] previously prepared 27 rebar samples of varying diameters with significant pitting morphologies using a half-immersion and electrification-accelerated corrosion method in the laboratory. All rebar samples were scanned using a 3D scanner to reconstruct their three-dimensional geometric models. These models were then imported into the parametric software PTC Creo 10 and sliced along the length of the rebar at 1 mm intervals. This procedure provided the cross-sectional shapes and the distribution of the cross-sectional areas of the corroded rebar at 1 mm intervals, with Figure 2 depicting the area distribution for the rebar with an 8 mm diameter. The distribution data revealed distinct localized pitting in the 8 mm corroded rebar.

**Figure 3:**
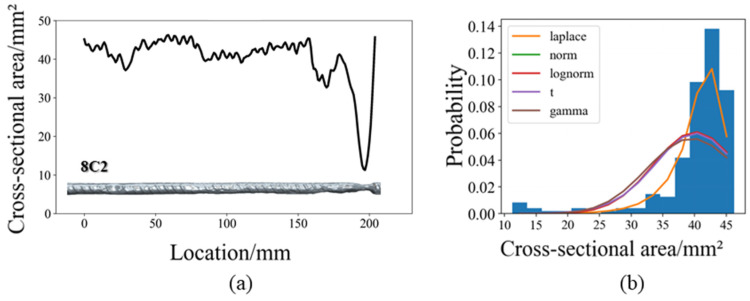
Fitting analysis: (**a**) the corrosion distribution along the rebar axis and (**b**) the histogram representing the residual cross-sectional area of the steel bars along with their corresponding fitted probability density curves [9].

Some studies have focused on developing probabilistic distribution models for the cross-sectional area of corroded reinforcements. These models are derived by fitting the cross-sectional data of the corroded reinforcement to determine an optimal probability density function [53]. Common probability density functions include the normal distribution [52], log-normal distribution [53], sinusoidal distribution [50], and Gumbel extreme value distribution functions [54,55]. However, in the fitting process, traditional unimodal distribution functions tend to shift their peak towards the main portion of the cross-sectional distribution to minimize error. As a result, the sections with smaller cross-sectional distributions, particularly those with pitting corrosion, receive lower probability density values and are often overlooked in the fitting process, as illustrated in Figure 3.

However, the mechanical properties of reinforcements are typically governed by their minimum cross-sectional area. Thus, underestimating the probability density of this portion can result in an overestimation of the load-bearing capacity and deformation ability of the corroded reinforcement. Clearly, unimodal probability distribution function models are not suitable for corroded reinforcements with significant pits. Therefore, this study employs the Gaussian Mixture Model (GMM) to accurately capture the cross-sectional distribution characteristics of pitted reinforcements. The GMM is derived from a weighted combination of multiple Gaussian (normal) distributions, and its probability density function is given by the following [9]:

(1) $$f(x,w_{k},\mu_{k},\sigma_{k})=\sum_{k=1}^{M}\frac{w_{k}}{\sigma_{k}\sqrt{2\pi }}\cdot e^{-\frac{{\left(x-\mu_{k}\right)}^{2}}{2\sigma_{k}^{2}}}$$

where the parameter *x* represents the value of the random variable; *n* denotes the number of clusters in the Gaussian Mixture Model; *μ_k_* and *σ_k_* represent the mean and variance of the *k*-th normal distribution, respectively; and *w_k_* is the weight coefficient of the random variable *x* attributed to the *k*-th normal distribution, indicating the relative proportion of the observed sample belonging to the cluster represented by the *k*-th normal distribution. The weights must satisfy the conditions 0 < *w_k_* < 1 and *w_1_* + *w_2_* + … + *w_M_* = 1.

By applying the Expectation–Maximization (EM) algorithm [56] to perform a cluster analysis on the cross-sectional area distribution of pitted reinforcements, it was found that a dual-cluster Gaussian Mixture Model (GMM) effectively describes the cross-sectional area distribution of most pitting corroded reinforcements [9]. The dual-cluster GMM possesses a clearer physical significance: the Gaussian component with a larger weight and higher mean corresponds to the cross-sectional area distribution of the reinforcement segments that exhibit more uniform corrosion, whereas the other Gaussian component with a smaller weight and lower mean corresponds to the segments where significant pitting occurs.

**Figure 4:**
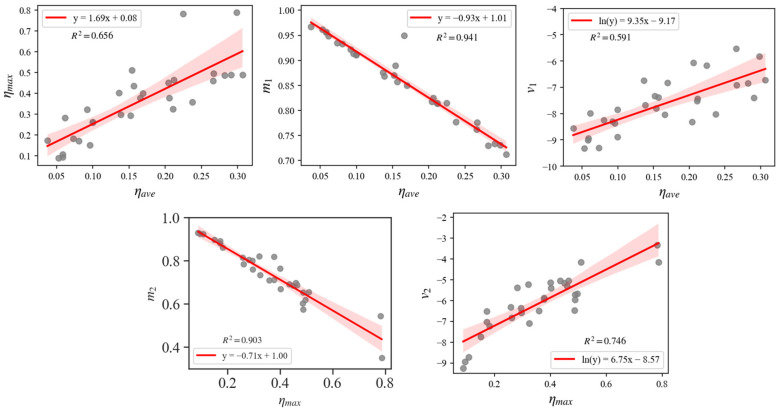
The regression analysis results of the dual-cluster GMM parameters [9].

In this way, a direct relationship can be established between the six parameters of the dual-cluster GMM and the morphological features of the corroded reinforcement. Specifically, the two weight coefficients, *w_1_* and *w_2_*, are related to the proportion of the pitted segment along the entire length of the reinforcement. The two mean coefficients, *m_1_* and *m_2_*, represent the average corrosion level *η_ave_* and the maximum cross-sectional corrosion level *η_max_* of the reinforcement, respectively. The two variance coefficients, *v_1_* and *v_2_*, define the distribution’s specific shape. The EM algorithm can be used to determine the dual-cluster GMM parameter values for various corroded rebars. By treating *η_ave_* and *η_max_* as independent variables, and *m_1_*, *v_1_*, *m_2_*, and *v_2_* as dependent variables, a regression analysis was performed using the least squares method. The regression analysis results for these parameters, obtained from 27 corroded steel bars, are presented in Figure 4. In the figure, the red line represents the regression equation, while the red translucent band indicates the confidence interval. The resulting regression equations for these parameters are provided in Table 1.

### 2.2. Degraded Model for Non-Uniform Corroded Reinforcement

**Figure 5:**
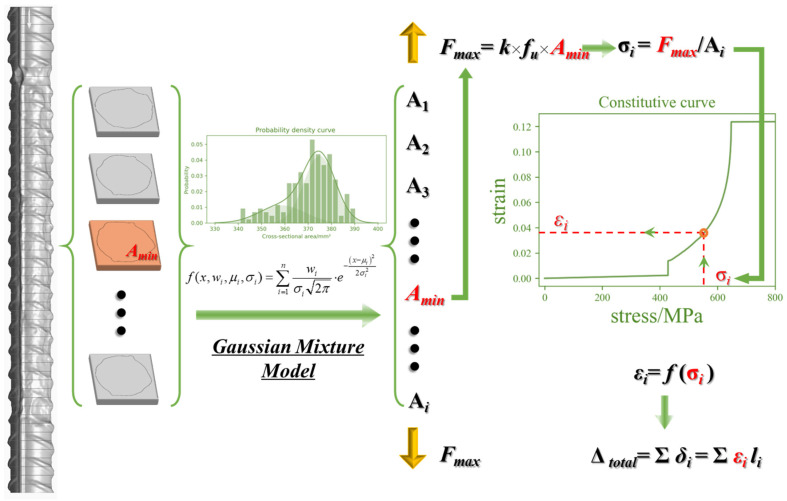
The computational procedure for the micro-segment deformation accumulation method.

Extensive experimental studies have consistently shown that the ultimate load-bearing capacity of corroded reinforcements is primarily governed by their minimum residual cross-sectional area [9,10,11,12,46,50], with little difference observed in the effective strength between corroded and uncorroded rebar. Based on the assumption that the original stress–strain behaviour of the material in corroded rebar remains unchanged, a numerical tensile testing method, as proposed in [10,57], effectively describes the load-displacement curve of corroded rebar. The detailed computational procedure is shown in Figure 5. Using the minimum cross-sectional area of the corroded reinforcement and the ultimate stress of the original reinforcement, the ultimate load for the corroded reinforcement can be determined. The deformation of all cross-sections is then integrated to calculate the ultimate elongation of the corroded reinforcement. The reliability of this numerical method has been validated by [9,10] through comparisons with tensile test results for corroded reinforcements and Digital Image Correlation (DIC) deformation fields on the reinforcement surface at the point of maximum load.

### 2.3. Tension Stiffening Effect in Reinforced Concrete

Stirrups, when embedded in concrete, are influenced by bond transfers at the rebar–concrete interface, leading to variations in local tensile stresses within the concrete. These stresses can partially reduce the tensile load carried by the reinforcement. The distribution of local stresses in the concrete varies along the axis of the rebar, peaking at the center of the micro-segments and decreasing to a minimum at the crack locations. To accurately assess the tensile mechanical properties of stirrups within concrete, it is essential to account for the impact of the tension stiffening effect in reinforced concrete.

This study models the tensile performance of concrete after cracking based on the one-dimensional uniaxial pullout test results for RC elements by Shima et al. [58]. The strength of concrete after tension stiffening can be represented using the following equation:

(2) $$\sigma_{t}=f_{t}{\left(\frac{\varepsilon_{tu}}{\varepsilon_{t}}\right)}^{c}$$

In Equation (2), *σ_t_* represents the tensile stress, *f_t_* is the tensile strength of the concrete, *ε_tu_* denotes the concrete cracking strain, *c* is the enhancement factor, typically taken as 0.4, and *ε_t_* represents the average tensile strain.

The tension stiffening effect induced by the presence of stirrups extends over a certain range, which can be calculated based on the cross-sectional area of the stirrups. The side length of the tension stiffening region in the concrete *R*_max_ is given by the following:

(3) $$R_{\max}=2\times \sqrt{\frac{1}{\pi }\times As\times \frac{f_{y}}{f_{t}}}$$

where *A_S_* represents the cross-sectional area of the stirrups, and fy denotes the yield strength of the stirrups.

Building on the preliminary assessment of the mechanical performance of corroded stirrups in Section 1, the ultimate strain of the corroded reinforcement was used as the strain *ε_t_* for concrete. Substituting this into Equations (2) and (3) allows for the determination of the additional strength provided by the concrete to the tensile stirrups. Using the numerical tensile testing method, the ultimate load capacity and updated deformation capacity of the corroded stirrups were re-estimated.

## 3. Impact of Corroded Stirrups on Core Concrete

As reported in the previous experimental studies on the mechanical performance of corroded rebar [9,10,12], uniform corrosion caused by carbonation generally results in a linear degradation of load-bearing capacity, while the deformation capacity would not be negatively affected. Accordingly, for “uniform corrosion” cases, the deformation capacity is not expected to differ significantly from that of a non-corroded specimen, apart from the reduction in load-bearing capacity. In contrast, pitting corrosion induced by chloride exposure leads to a sharp decline in the deformation capacity of the reinforcement. It is therefore essential to reasonably account for the degradation of the confinement effect on core concrete caused by pitting stirrups. The impact of pitting stirrups on the confinement effect of core concrete can be determined by integrating the residual performance of corroded stirrups, as calculated in Section 2, into the existing constitutive studies of confined concrete by Manda et al. [23] and Vu et al. [40]. Specifically, the peak stress and strain of concrete that is influenced by corroded stirrups can be described as follows:

(4) $$f_{cc}^{\prime }=(1-\alpha \eta_{ave})f_{c0}^{\prime }(-1.254+2.254\sqrt{1+\frac{7.94f_{l}^{\prime }}{f_{c0}^{\prime }}}-2\frac{f_{l}^{\prime }}{f_{c0}^{\prime }})$$

(5) $$\dot{\varepsilon_{cc}}=(1-\beta \eta_{ave})\varepsilon_{c0}\left[1+5(\frac{f_{cc}^{\prime }}{f_{c0}^{\prime }}-1)\right]$$

where *η*_ave_ is the average corrosion rate of the stirrups; *f′_cc_* and *ε′_cc_* are the corrected peak stress and strain of the concrete confined by corroded stirrups; *f′_c0_* and *ε′_c0_* represent the peak stress and strain of unconfined concrete; *α* and *β* are the degradation factors for the peak stress and strain of unconfined concrete, respectively, with values of 0.19 and 0.49 for square sections, and 0.51 and 0.28 for circular sections [40]; and *f′_l_* is the effective lateral confining pressure exerted by the corroded stirrups on the core concrete. To consider the impact of pitting corrosion on the confinement provided by corroded stirrups, this study defines the effective lateral confining pressure as follows:

(6) $$f_{l}^{\prime }=\frac{1}{2}k_{e}(1-\eta_{ave})\rho_{s}f_{yhc}$$

where *ρ_S_* is the volumetric ratio of the stirrups before corrosion; *f_yhc_* is the corrected yield strength of the stirrups after corrosion; and *k_e_* is the confinement effectiveness coefficient. For rectangular and circular sections confined by stirrups, the calculation of *k_e_* follows the formula proposed by Mander et al. [23], which is expressed as follows:

(7) $$k_{e}=\frac{\left(1-\sum_{i=1}^{n}\frac{{\left(w_{i}^{\prime }\right)}^{2}}{6b_{c}d_{c}}\right)\left(1-\frac{s^{\prime }}{2b_{c}}\right)\left(1-\frac{s^{\prime }}{2d_{c}}\right)}{\left(1-\rho_{cc}\right)}$$

(8) $$k_{e}=\frac{1-\frac{s^{\prime }}{2d_{s}}}{\left(1-\rho_{cc}\right)}$$

In these equations, *b_c_* and *d_c_* are the width and height of the core concrete, respectively; *w _i_*′ is the clear spacing between longitudinal bars; *S*′ is the clear spacing between stirrups; *ρ_cc_* is the longitudinal reinforcement ratio; and ds represents the clear diameter between the centers of the spiral stirrups.

As the damage from stirrup corrosion continues to accumulate, the material properties of the stirrups progressively degrade, leading to a reduction in their confining effect on the concrete. Consequently, the ultimate strain of the confined concrete correspondingly reduces. In this study, the strain corresponding to the failure of the first confining stirrup in the reinforced concrete components was taken as the ultimate strain of the concrete. To evaluate the ultimate strain of concrete reinforced with corroded stirrups, a modification of Paulay and Priestley’s [59] empirical formula was conducted by incorporating a reinforcement damage model that accounts for pitting effects. The derived formula for the ultimate strain of confined concrete is presented below:

(9) $$\varepsilon_{cu}^{\prime }=0.004+\frac{1.4(1-\eta_{\alpha ve})\rho_{s}f_{yhc}\varepsilon_{smc}}{f_{cc}^{\prime }}$$

where *ε′_cu_* represents the corrected ultimate strain of the concrete confined by corroded stirrups, and *ε_smc_* denotes the tensile strain corresponding to the peak tensile stress in the corroded stirrups.

After adjusting the key parameters for the core concrete, the complete stress–strain curve for the core concrete can be calculated using Equations (10) and (11):

(10) $$f_{c}=\frac{f_{cc}^{\prime }(\varepsilon_{c}/\varepsilon_{cc}^{\prime })r}{r-1+{\left(\varepsilon_{c}/\varepsilon_{cc}^{\prime }\right)}^{r}}$$

(11) $$r=\frac{5000\sqrt{f_{c0}^{\prime }}}{5000\sqrt{f_{c0}^{\prime }}-f_{cc}^{\prime }/\varepsilon_{cc}^{\prime }}$$

where *f_c_* and *ε*_c_ represent the stress and strain values of the confined concrete, respectively, and r is the shape factor of the curve. Equations (10) and (11) provide a method to calculate of the stress corresponding to each strain value for core concrete under the different corrosion levels of the stirrup.

It is worth noting that in certain existing structures, such as old buildings designed with inadequate seismic details or bridge piers constructed more than 50 years ago, large stirrup spacings (typically 20–30 cm) are commonly observed [34]. Under such conditions, the confinement action provided by transverse reinforcements is inherently modest, even in uncorroded scenarios. Consequently, the impact of corrosion on the confinement effect of concrete may play a relatively minor role, and the confinement provided by the stirrups in such cases can conservatively be considered negligible.

## 4. Validation of Effectiveness

**Figure 6:**
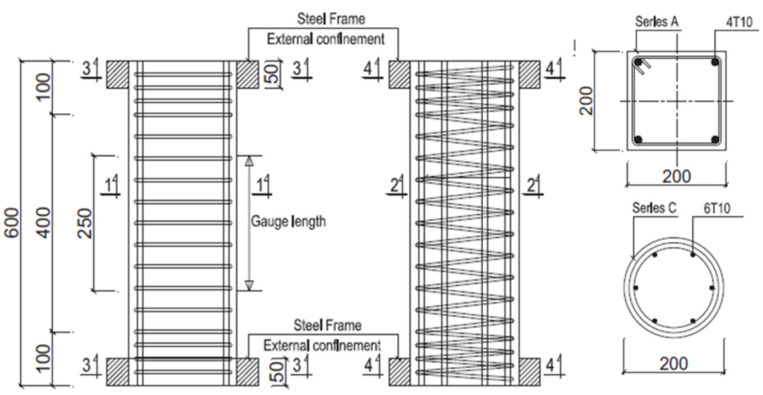
Section dimensions and reinforcement retails (mm) [40].

To verify the accuracy and applicability of the revised model presented in this study, 12 axially loaded reinforced concrete column specimens designed by Vu et al. [40] were selected for analysis and validation. These specimens have a concrete cover of 10 mm and include two types of cross-sectional shapes: rectangular columns (Type A) and circular columns (Type C). Figure 6 depicts the shapes and dimensions of the cross-sections. For each cross-sectional type, the RC columns were constructed with three different stirrup spacings, labelled as L, M, and S to represent large, medium, and small spacings, respectively. Each stirrup spacing configuration included columns with two different levels of corrosion rates. The detailed corrosion information is presented in Table 2.

**Figure 7:**
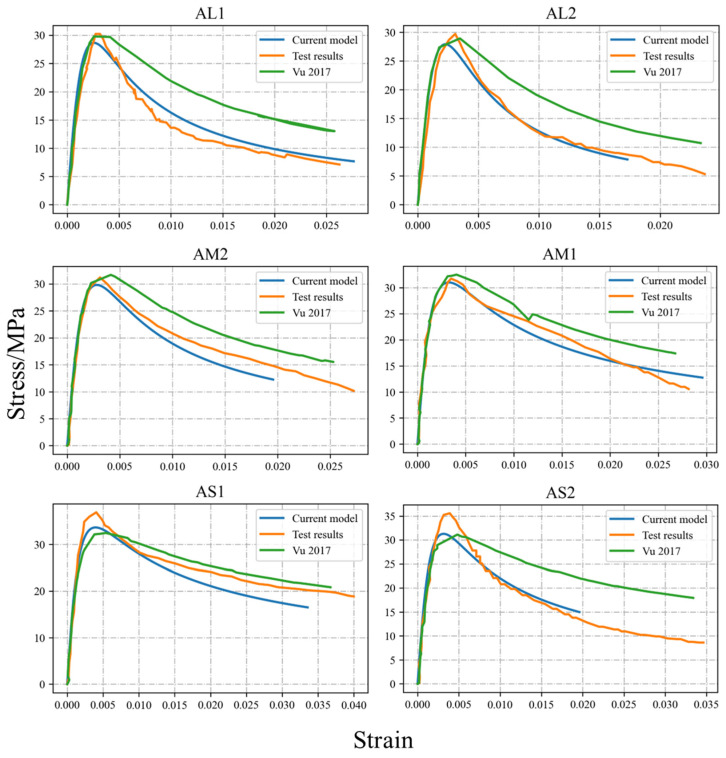
Comparison between test results [40] and calculation results for rectangular section specimens.

**Figure 8:**
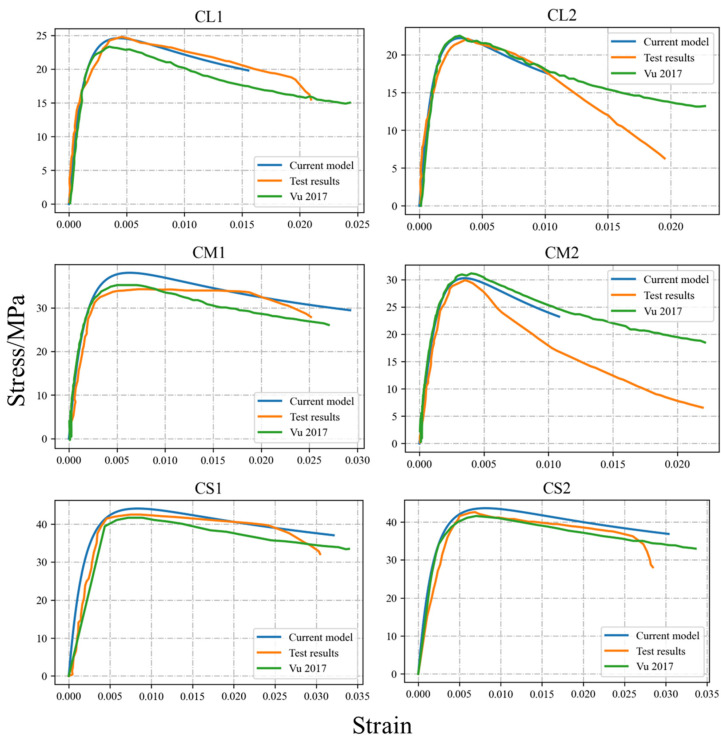
Comparison between test results [40] and calculation results for circular section specimens.

Since the Vu et al. [40] model calculates the level of reinforcement corrosion based on the assumption of uniform corrosion, the previous study only provided the *η_ave_* of the stirrups. According to the regression relationship between the *η_max_* and *η_ave_* of the pitting corroded rebars listed in Table 1, the *η_max_* can be determined. The potential cross-sectional data with the corresponding distribution characteristics can be generated through the GMM sectional distribution probability model provided in Table 1. The initial mechanical properties of the stirrups were calculated using the method proposed in Section 2.2. Then, Equations (2) and (3) were used to incorporate the tension stiffening effect in reinforced concrete, further refining the mechanical behaviors of the corroded stirrups. Finally, the revised mechanical behaviors were input into Equations (10) and (11) to determine the stress–strain curves of the confined concrete. These curves were then compared with those from the Vu et al. model and the original experimental curves, as illustrated in Figure 7 and Figure 8.

By comparing the experimental results with the predictions from the Vu et al. model and the theoretical model proposed in this study, it is evident that all three stress–strain curves align well at lower levels of average corrosion, demonstrating their reliability under minimal deterioration. However, when the effects of stirrup pitting are considered, the revised model proposed here achieves a significantly closer match with the experimental curves than the Vu et al. model, which assumes uniform corrosion and does not account for the non-uniform degradation caused by pitting.

At higher corrosion levels, for rectangular sections, the Vu et al. model shows a noticeable degree of error when compared to the experimental data. This discrepancy highlights the limitations of assuming uniform corrosion in accurately reflecting the mechanical behaviors of corroded stirrups. In contrast, the stress–strain model revised in this study effectively incorporates the impact of pitting, providing more accurate predictions that align closely with experimental results. For circular sections, however, the revised model tends to conservatively estimate the deformation capacity of the core concrete. This conservative behaviour can be attributed to the superior confinement effect of spiral stirrups and their small spacing, which prevent immediate crushing of the core concrete even when stirrup fractures occur during testing.

Overall, the revised theoretical model successfully captures the constitutive relationships of core concrete across various cross-sectional shapes, corrosion levels, and stirrup ratios. Its ability to account for non-uniform pitting corrosion and the resultant mechanical degradation offers a more robust and realistic framework for predicting the performance of confined concrete under diverse conditions.

## 5. Lifetime Stress–Strain Model Prediction for Concrete Confined by Pitted Stirrups

**Figure 9:**
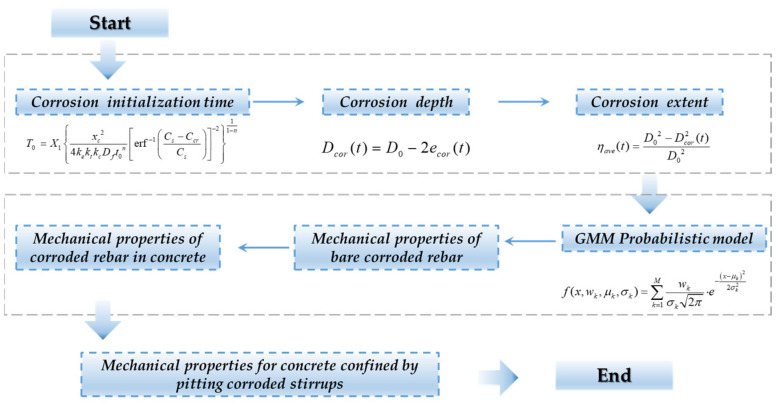
Flowchart of lifetime mechanical properties for concrete confined by pitted stirrups.

In this section, a demonstrative example is provided to illustrate the complete workflow of the proposed method for assessing the residual mechanical performance of core concrete in practical engineering applications. The detailed computational procedure is depicted in Figure 9. The prototype for this example is based on the AL-series specimens from the experimental study conducted by Vu et al. [40].

**Figure 10:**
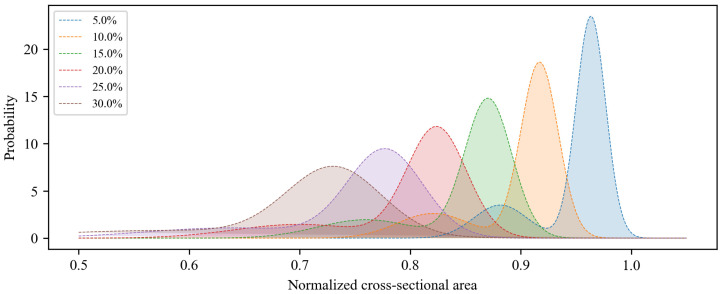
Time-variant probabilistic density curves of GMM.

The procedure begins with the evaluation of the corrosion level of the structure. According to current research findings, the initiation time of the corrosion, corrosion depth, and average corrosion rate can be effectively determined based on environmental conditions such as the humidity, temperature, and chloride ion concentration. Detailed explanations of these evaluation models can be found in references [60,61]. The proposed model in this study is developed under the assumption that *ηave* is known. Subsequently, based on the regression equations provided in Figure 4 and Table 1, a two-component GMM of the residual cross-sectional area for pitting reinforcements can be established. Following the suggestion in [8], this study adopts weights *w_1_ = 0.79* and *w_2_* = 0.21. The GMM probability density curves were plotted for average corrosion rates ranging from 5% to 30%, as illustrated in Figure 10.

**Figure 11:**
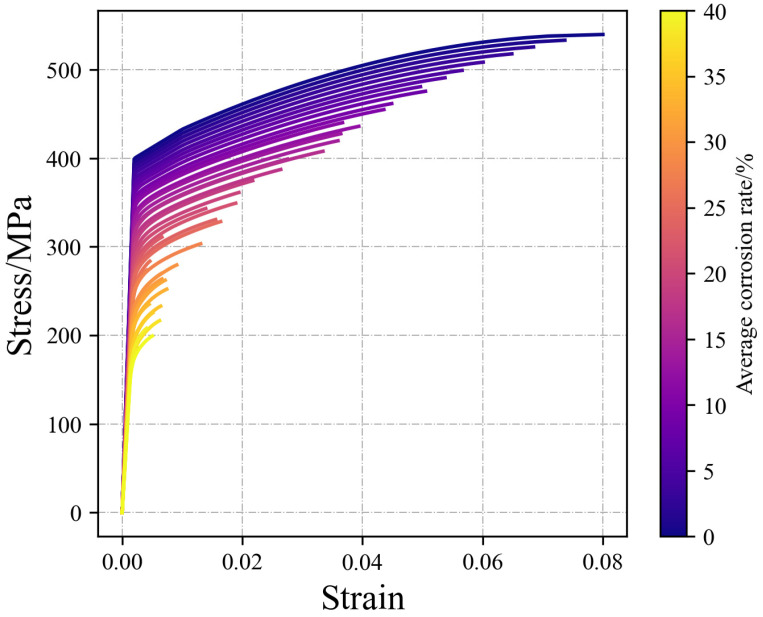
Time-variant stress–strain curves of corroded rebars.

As discussed in Section 2.2, once the GMM model for the residual cross-sectional distribution of reinforcements is obtained, mathematical analysis software such as Python 3.8 or MATLAB 2020 can be utilized to randomly generate residual cross-sectional area data corresponding to various corrosion degrees. Combined with numerical tensile testing, a time-dependent degraded model for reinforcements under any given corrosion level can be obtained, as illustrated in Figure 11.

**Figure 12:**
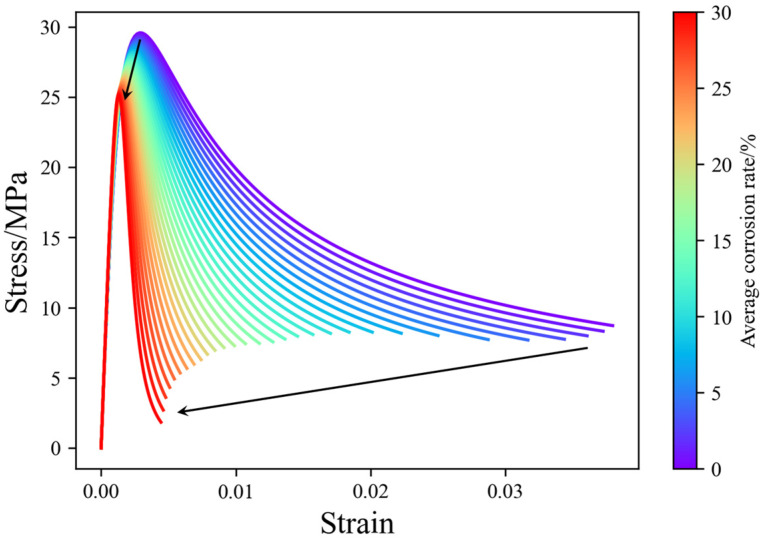
Time-dependent damage curves for concrete confined by pitting corroded stirrups.

After determining the initial mechanical properties of the stirrups, Equations (2) and (3) were applied to account for the tension stiffening effect in reinforced concrete. These refined properties were then utilized in Equations (10) and (11) to derive the stress–strain curves for the confined concrete. Figure 12 illustrates the stress–strain curves of confined concrete under different stirrup corrosion levels.

With increasing corrosion, the non-uniform corrosion of the stirrups has a limited impact on the peak stress and corresponding strain of the core concrete but significantly reduces its ultimate strain. This effect occurs because corrosion causes a linear reduction in the load-bearing capacity of the reinforcement, while its impact on deformation capacity is nearly exponential. As a result, the deformation capacity of the core concrete declines sharply, accompanied by a continuous weakening of its confining effect on the surrounding confined concrete.

## 6. Conclusions

This study builds upon existing research on corroded reinforcements to develop a probabilistic model for the residual cross-sectional area distribution, providing a reasonable assessment of the impact of pitting corrosion on the mechanical behaviors of stirrups. By considering the tension stiffening effect in reinforced concrete, we have further revised existing stress–strain models for concrete confined by stirrups. Our comparisons with previous experimental results and theoretical models have led to the following conclusions:

(1) Compared to traditional unimodal distribution models, the Gaussian Mixture Model (GMM) provides an excellent representation of the residual cross-sectional distribution characteristics of pitting corroded rebars. Furthermore, for most pitting corroded rebars, the GMM can be simplified into a dual-cluster model. The primary Gaussian component with the larger weight reflects the cross-sectional area distribution of the uniformly corroded segments, while the secondary Gaussian component with a lower weight and mean captures the distribution characteristics of the pitted segments.
(2) Due to the bond effect between the concrete and reinforcement, the tension stiffening effect of the concrete significantly influences the tensile mechanical performance of the corroded reinforcement. This influence cannot be overlooked. The tensile test results of individual corroded rebars can be adjusted by incorporating an equivalent load increase, thus allowing an accurate assessment of their tensile mechanical performance within concrete.
(3) The stress–strain model proposed in this study for concrete confined by corroded stirrups aligns more closely with the experimental results than previous research. It effectively reflects the degradation in the mechanical performance of concrete confined by corroded stirrups. Moreover, the presence of pitting corrosion in stirrups accelerates the deterioration of their performance. Continuing to use a degradation analysis under the assumption of uniform corrosion is likely to result in an overestimation of the mechanical performance of corroded RC components.

This study lays a meaningful groundwork for understanding the impact of pitting corrosion on the mechanical performance of stirrups and confined concrete. While the proposed model demonstrates its effectiveness, future research will focus on experimental validation to further enhance its reliability and applicability. Additionally, the Gaussian Mixture Model (GMM), though sophisticated, serves as a valuable tool for analyzing non-uniform corrosion characteristics. Future efforts will include large-scale finite element analyses and experimental studies to better quantify the influence of pitting corrosion on RC component performance.

## Figures and Tables

**Table 1:** The regression equations for the GMM parameters.

Parameters/*y*	Arguments/*x*	Regression Equation
*η_max_*	*η_ave_*	*y* = 1.69*x* + 0.08
*m_1_*	*η_ave_*	*y* = −0.93*x* + 1.01
*v_1_*	*η_ave_*	*y* = e^(9.35*x* − 9.17)^
*m_2_*	*η_max_*	*y* = −0.71*x* + 1.00
*v_2_*	*η_max_*	*y*= e^(6.75*x* − 8.57)^

**Table 2:** Parameters related to stirrup corrosion and confined concrete (units: MPa and mm).

Specimen	Concrete			Longitudinal Rebar			Stirrup				
	*f^′^_c_*	*f^′^*_c0_	*ε* ^′^ *_c_*_0_	Number	*D_ll_*	*f_y_*	*D_h_*	Space	*f_yh_*	*ρs*/%	*η_ave_*/%
AL1	30.5	25.4	0.0016	4	10	568	6	65	360	0.97	4.9
AL2	30.5	25.4	0.0016	4	10	568	6	65	360	0.97	9.8
AM1	30.5	25.4	0.0016	4	10	568	6	40	360	1.57	7.3
AM2	30.5	25.4	0.0016	4	10	568	6	40	360	1.57	12.5
AS1	29.3	24.9	0.0015	4	10	568	6	25	360	2.51	9.5
AS2	29.3	24.9	0.0015	4	10	568	6	25	360	2.51	16.7
CL1	25.3	18	0.0016	6	10	568	6	55	360	1.14	16.8
CL2	25.3	18	0.0016	6	10	568	6	55	360	1.14	21.8
CM1	31.9	24.4	0.0017	6	10	568	6	40	360	1.57	7.6
CM2	31.9	24.4	0.0017	6	10	568	6	40	360	1.57	22.8
CS1	31.9	24.4	0.0017	6	10	568	6	25	360	2.51	9.3
CS2	31.9	24.4	0.0017	6	10	568	6	25	360	2.51	10

## Data Availability

The original contributions presented in this study are included in the article, and further inquiries can be directed to the corresponding author.
